# Mechanical Characterization of Multifunctional Metal-Coated Polymer Lattice Structures

**DOI:** 10.3390/ma17030741

**Published:** 2024-02-03

**Authors:** Lizhe Wang, Liu He, Fuyuan Liu, Hang Yuan, Ji Li, Min Chen

**Affiliations:** 1 School of Advanced Technology, Xi’an Jiaotong-Liverpool University, Suzhou 210053, China; 2 School of Engineering, University of Liverpool, Liverpool L69 3BX, UK; 3 Key Laboratory of MEMS of the Ministry of Education, Southeast University, Nanjing 210096, Chinaj.li5@seu.edu.cn (J.L.)

**Keywords:** body-centered cubic lattice, multiscale analysis, elastic tensile stiffness, elastic bending stiffness, copper-coated lattice

## Abstract

Metal-coated lattice structures hold significant promise for customizing mechanical properties in diverse industrial applications, including the mechanical arms of unmanned aerial vehicles. However, their intricate geometries pose computational challenges, resulting in time-intensive and costly numerical evaluations. This study introduces a parameterization-based multiscale method to analyze body-centered cubic lattice structures with metal coatings. We establish the validity and precision of our proposed method with a comparative analysis of numerical results at the Representative Volume Element (RVE) scale and experimental findings, specifically addressing both elastic tensile and bending stiffness. Furthermore, we showcase the method’s accuracy in interpreting the bending stiffness of coated lattice structures using a homogenized material-based solid model, underscoring its effectiveness in predicting the elastic properties of such structures. In exploring the mechanical characterization of coated lattice structures, we unveil positive correlations between elastic tensile stiffness and both coating thickness and strut diameter. Additionally, the metal coating significantly enhances the structural elastic bending stiffness multiple times over. The diverse failure patterns observed in coated lattices under tensile and bending loads primarily stem from varied loading-induced stress states rather than external factors. This work not only mitigates computational challenges but also successfully bridges the gap between mesoscale RVE mechanical properties and those at the global structural scale.

## 1. Introduction

Lattice structures are of great interest in various fields due to their unconventional mechanical properties, such as high specific stiffness [1], excellent energy absorption capability [2], and negative Poisson’s ratio [3,4]. These structures have found applications in the manufacturing processes of angioplasty stents [5,6], bone tissue engineering [7], and cranial remodeling orthosis [8]. With the advent of three-dimensional (3D) printing technology, the design of lattice structures has become increasingly diverse, enabling the fabrication of complex components using highly automated processes [9,10,11]. Typically, flexible and non-toxic biocompatible polymers [12], such as resin and polyurethane, are used to manufacture lattice structures, but they often lack sufficient stiffness and strength. To address this limitation, metal-based additive manufacturing (AM) has been used to fabricate lattice metamaterials using metals such as copper alloy and titanium alloy [13,14]. Although some structures having both resilience and stiffness were fabricated, the geometrical and application restrictions were extremely rigorous [15].

Presently, the simultaneous improvement in multiple mechanical properties in lattice structures composed of a single material poses a significant challenge [16,17]. Consequently, there is a growing focus among scholars on composing lattices using diverse materials and reinforcement strategies, offering advantages such as enhanced strength and energy absorption [18,19,20]. The combination of the stability and shape recoverability after deformation of polymetric cores, along with the overall mechanical performance provided by metallic coating shells [21,22,23] (e.g., high plasticity-induced deformation limitation, impressive stiffness, strength, and conduction properties of copper material), underscores the considerable potential of AM-based metal-coated lattices across various industrial applications. In addition to overcoming the weaknesses of individual materials and yielding exceptional mechanical properties [24,25,26], scholars have extensively explored the tunable mechanical properties of lattice metamaterials. For instance, Kao et al. [27] verified the high cell adhesion capacity of coated 3D-printed scaffolds for bone tissue replicas, and Xiao et al. [28] proposed a titanium-coated lattice structure for mandibular prosthesis to improve compressive strength and biocompatibility. Finite element (FE) definition methods have been analyzed for appropriate simulation due to the micron-sized dimension of the coating [29,30], and coating layer performance and contribution to general mechanical properties have been studied analytically [31], numerically [32,33], and experimentally [34]. Song et al. [35] analyzed the structural response of a nickel-coated body-centered cubic (BCC) lattice structure, which showed a 68% effective elastic modulus compared with pure polymer lattices. However, due to the diverse loading conditions that lattice structures can encounter, understanding the impacts of micro/mesoscopic scale-based design factors on macroscopic structural capacities is crucial. Despite this, global level-based mechanical property studies of lattice structures are resource-intensive, and peculiar lattice characteristics require strict requirements for element discretization and numerical calculation, limiting research on the impacts of coated lattice design parameters on entire structural properties.

Multiscale analysis, serving as a bridge between global and local perspectives, has proven to be effective for studying the mechanics of various structures with symmetry and periodicity, such as composites [36], hollow structures [37], and thin-walled pressure-bearing structures [38]. By using the volumetric averaging method and constitutive relations based on homogenization theory [39,40], multiscale analysis anticipates structural properties. The Representative Volume Element (RVE), which is the minimal repeatable microscale structure for homogenization, enables large-scale structure analysis at limited computation costs. Lattice structures, comprising solid rods and voids, are a special type of composites, and the rationality of mapping local properties to the equivalent global level by multiscale analysis has been validated from this perspective [41]. However, most multiscale studies only consider the geometry compatibility of the single geometric cell as the RVE and neglect the impact of the RVE size on the structure. Despite research on homogeneous lattice structures, little attention has been paid to numerically predicting the material properties of metal-coated lattices.

**Figure 1:**
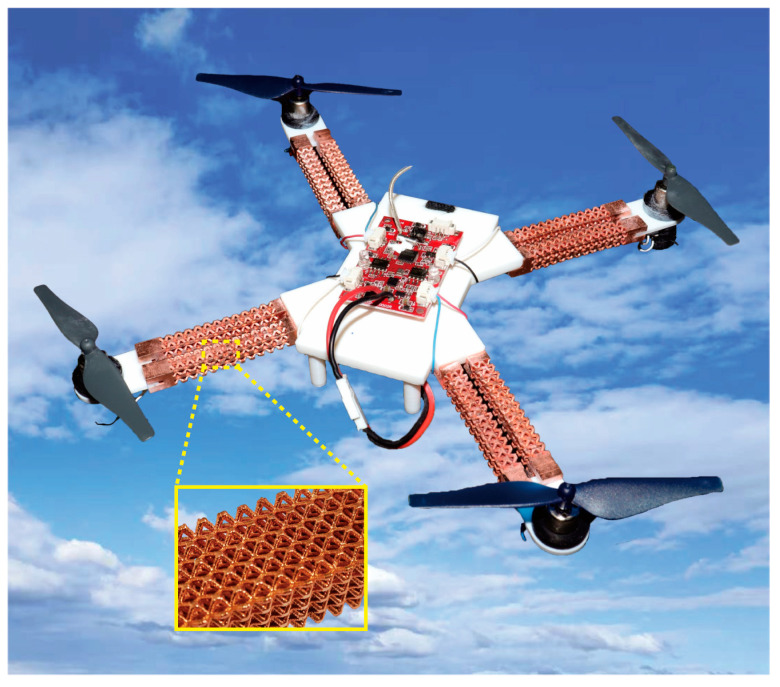
The schematic of the UAV design featuring the lattice framework arm [42].

Due to their exceptional lightweight performance, discontinuous fracture mechanism, and the metal-based aging retardation of the photopolymer matrix, we have successfully implemented metal-coated BCC lattice structures in a representative application—specifically, the frame arms of unmanned aerial vehicles (UAVs), as illustrated in Figure 1 [42]. In this context, we conduct mechanical characterizations of multifunctional BCC copper-coated lattice structures using a multiscale analysis method. This approach accounts for the influence of lattice cell numbers within a single RVE. The structure of this study unfolds as follows: Section 2 outlines the material and model preparations for both macroscopic experiments and numerical calculations. Additionally, we propose a numerical method to anticipate the mechanical properties of coated lattices using a multiscale analysis based on periodic boundary conditions (PBCs). Moving on to Section 3, we validate the rationality and precision of our proposed method with a comprehensive comparison of simulation and experimental results. Furthermore, we uncover the impacts of configuration design and coating film on the structural elastic tensile and bending properties.

## 2. Materials and Methods

### 2.1. Sample Design and Experimental Test

#### 2.1.1. Specimen Design and Manufacturing

**Figure 2:**
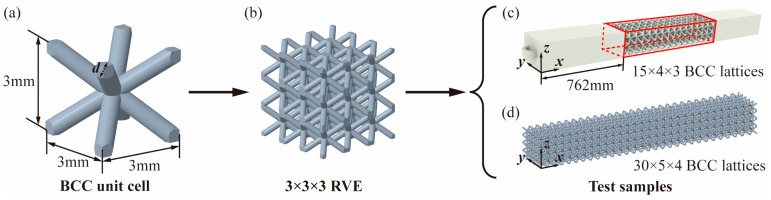
The process flowchart of sample modeling, initiating with (**a**) the single lattice cell, progressing to (**b**) the cell array, and culminating in the final specimens for the (**c**) tensile and (**d**) bending tests.

Each lattice cell was designed with dimensions of 3 mm × 3 mm × 3 mm using Creo 8.0, as depicted in Figure 2a. To ensure both feasibility and cost-effectiveness in 3D printing lattice structures consisting of unit cells with this configuration, the permissible rod diameter d was designed to range from 0.6 mm to 1.5 mm. In order to thoroughly investigate the rationality and applicability of the proposed numerical method, BCC lattices with d values of 0.6 mm and 1 mm were printed for the subsequent sections, focusing on numerical method validation and characterization interpretations. Utilizing these designated parameters, lattice structures with multiple cells were created in an array, as depicted in Figure 2b, constituting the final specimen models for experimental tests. Specifically, sample models for both tensile and bending tests were established with a framework of 15 × 4 × 3 and 30 × 5 × 4 in the x, y, and z directions, respectively, as illustrated in Figure 2c,d.

**Figure 3:**
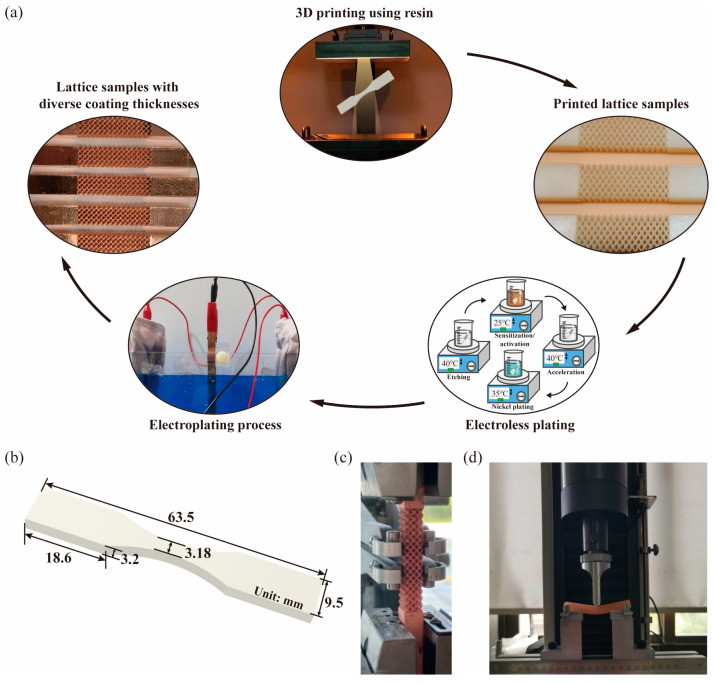
Integrated manufacturing processes and property characterization experiments, featuring (**a**) the comprehensive fabrication steps for copper-coated lattice samples, (**b**) dimensions of the dog-bone samples, (**c**) quasi-static in situ tensile tests, and (**d**) three-point bending tests for the copper-coated lattice samples.

Subsequently, a high-precise stereolithography appearance (SLA) 3D printer (Lite 600HD, UnionTech, Beijing, China, 355 nm) was used here to prepare the lattice bases made of photo-curable resin (SH8801, UnionTech). The critical print parameter, layer thickness, was set to 0.05 mm. Then, as recommended by UnionTech, the resin lattice specimens were washed with isopropanol (IPA) for 1 min (25 °C) and then were post-cured with a 355 nm UV light at 60 °C for 45 min durations. As electroplating involves the electrolysis principle to deposit metal layers on conductive surfaces, electroless plating was used in this study. It served to deposit a nickel conductive layer onto the insulating resin lattice matrix, harnessing the benefits of uniform coating thickness and robust solution dispersion capabilities associated with electroless plating [26,43]. This step was undertaken in preparation for the subsequent electroplating process. As mentioned by previous studies [21,35], the electroless plating process can be summarized into four major steps: (1) etching, (2) sensitization/activation, (3) acceleration, and (4) nickel plating. Based on our peers’ previous study focusing on the influence of etching and adhesion film [44], the electroless plating process in this study was carried out as follows: The lattice samples were coarsened in 200 g/L KOH at 40 °C to improve surface roughness and washed thoroughly with DI water. Subsequently, the samples were pre-soaked in an acidic environment with 15% HCl at 25 °C and then immersed in a palladium catalyst activator containing 60 g/L of NaCl, 230 mL/L of HCl, and 10 mL/L colloidal palladium 878 for 5 min at 25 °C. After rinsing with DI water, they were put into 10% HCl at 40 °C to fully expose palladium catalysts. Then, the samples were immersed in electroless nickel plating solution (20 g/L of NiSO_4_·7H_2_O, 30 g/L of NaH_2_PO_2_·H_2_O, and 10 g/L of Na_3_C_6_H_5_O_7_) at 35 °C for 15 min. In this context, the nickel conductive layer has a thickness of less than 0.5 μm, as established in our prior studies [23]. This thickness is considerably smaller when compared with the thickness of the copper coating film. Consequently, the adhesive impact of the nickel film can be considered negligible during the subsequent numerical analysis. With the nickel conductive layer, the copper layer could be electroplated on the lattice structures in a bath containing 200 g/L CuSO_4_·5H_2_O and 30 mL/L H_2_SO_4_, which was conducted in the Haring cell with anode and cathode. This way, the copper-coated samples with diverse coating thicknesses T could be acquired correspondingly. To evaluate the process consistency of SLA and electroplating, five samples were printed for each coating thickness group. The completed manufacturing processes are schematically illustrated in Figure 3a.

#### 2.1.2. Mechanical Property Test

To facilitate material configuration in simulations and establish a connection between the mechanical properties of the mesoscale RVE and the macroscale structure, we conducted tensile and bending tests following the ASTMD638-14 standard [45]. Initially, dog-bone group samples, as depicted in Figure 3b with specified dimensions, were printed for tensile tests to ascertain the equivalent Young’s modulus of the resin matrix $E_{resin}$. Subsequently, using the aforementioned electroplating technique, copper-coated lattice structures were printed with T varying from 10 μm to 80 μm and were categorized into tensile and bending groups. The corresponding tests were carried out as illustrated in Figure 3c,d. Notably, each group comprised 5 test samples, and all quasi-static tensile and bending tests were conducted with displacement control at a rate of 1.5 mm/min. 

Scholars have delved into the unique mechanical properties exhibited by metallic thin films, noting distinctions from their bulk counterparts. These disparities arise from factors such as microscopic thickness, micromachining methods, or specific microstructures [46,47,48]. However, due to the challenges posed by the extremely small T, using existing techniques or standards for precisely characterizing the mechanical properties of copper films becomes problematic. Traditional in situ tensile tests, commonly effective for larger materials, face significant hurdles when it comes to accurately assessing the equivalent Young’s modulus of the copper film ($E_{copper}$). To address this issue, we propose a reverse approach: drawing on insights from previous investigations into the mechanical properties of electrodeposited copper films [46,49], we numerically parameterize $E_{copper}$ within the range of 100 GPa to 110 GPa, guided by the actual electrodepositing current density of 2 A/dm^2^. For clarity, $E_{lattice}$ and $F_{lattice}$ of the subsequent sections denote the corresponding elastic modulus and external loads of the copper-coated lattice derived from the numerical method, while $E_{lattice}^{*}$ and $F_{lattice}^{*}$ represent their counterparts obtained from experimental tests. By comparing $E_{lattice}$ across various $E_{copper}$ values with $E_{lattice}^{*}$, we can retroactively determine the most appropriate value for $E_{copper}$. 

**Figure 4:**
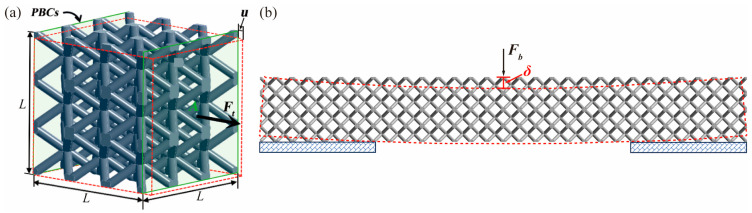
The schematic diagram illustrating the calculations of (**a**) $E_{lattice}$, and (**b**) $k_{b}$ for copper-coated BCC lattices.

The macroscopic behavior of the BCC structure can exhibit anisotropy, deviating from the isotropic nature of the bulk material due to preferential orientations resulting from the lattice RVE’s symmetry. However, under certain assumptions—namely, (1) elastic response in the RVE material and (2) neglecting structural buckling—the BCC lattice cell, following cubic syngony, assumes equal Young’s moduli along three orthogonal coordinate axes, denoted as $E_{1}=E_{2}=E_{3}$ [41,50]. With the conformity between the loading and geometrical directions of symmetry axes, the directional Young’s modulus ($E_{i}$, i=1,2,3) is regarded as equivalent to $E_{lattice}$ in this study. In this context, as schematically described in Figure 4, two types of stiffness, $E_{lattice}$ and the bending stiffness $k_{b}$, can be assessed. The detailed calculations are illustrated as follows:

(1) $$\left\{\begin{array}{c} E_{lattice}=\frac{\Sigma_{1}}{E_{1}}=\frac{F_{t}/A}{u/L}=\frac{F_{t}L}{uA}\\ k_{b}=\frac{F_{b}}{\delta } \end{array}\right..$$

where Σ and E denote the macroscopic stress and strain, respectively. $F_{t}$ is the uniaxial reaction force caused by the input tensile loading, u is the uniaxial external displacement, L and A represent the dimension and cross-sectional area of the lattice cell, $F_{b}$ stands for the external bending load, and δ is the deflection induced by $F_{b}$. 

### 2.2. Numerical Implementation

#### 2.2.1. Multiscale Evaluation Based on PBCs

The homogenization theory, relying on PBCs, assesses volume-weighted values for stress and strain. Scholars have widely used this approach to gain a comprehensive understanding of various lattice structures [51,52]. At its core, this theory posits that homogenized physical properties can be understood as parameters of a homogeneous material, exhibiting an overall response “close” to that of the heterogeneous periodic material as the cell size approaches zero [39,53]. Building on the PBC application method to lattice structures proposed in our earlier work [54], the loadings on the RVE of the BCC lattice structure should mirror the mechanical loading conditions of stress or strain on a macroscopic scale. In practical terms, the macroscopic pressure loads (equivalent with Σ) are applied on the boundary, assuming that each RVE is subject to the local stress field $\sigma^{E}$ with the relationship $\left\langle \sigma^{E}\right\rangle =\Sigma$, where $\left\langle \cdot \right\rangle$ denotes the volume average operator. Additionally, the alignment of loading directions with symmetry axes results in u and strain ε on the periodic boundaries of the RVE with $\left\langle \varepsilon \left(x\right)\right\rangle =E$. Thus, detailed mapping relations between the macroscopic mechanical properties of metal-coated lattice structures and the microscopic properties of lattice RVEs can be established as follows:

(2) $$\left\{\begin{array}{c} \begin{array}{c} div\;\left(\sigma^{E}\right)=0\;\;\;\;\;\;\;\;\;\;\;\;\;\;\;\;in\;V_{\mathrm{RVE}}\\ \left\langle \varepsilon \right\rangle =E\\ \sigma^{E}=d:\varepsilon =d:E\;\;\;\;\;\;\;\;\;\;in\;V_{\mathrm{RVE}}\\ \left\langle \sigma^{E}\right\rangle =\Sigma \\ u^{s}\;\;\;\;\;\;\;\;\;\;\;\;\;\mathrm{symmetric}\;on\;{\partial V}_{\mathrm{RVE}} \end{array}\\ \tilde{u}=\tilde{\varepsilon }=0\;\;\;\;periodic\;on\;{\partial V}_{\mathrm{RVE}}\\ \sigma^{E}\cdot n\;\;\;\;\;\mathrm{anti-periodic}\;\mathrm{on}\;{\partial V}_{\mathrm{RVE}} \end{array}.\right.$$

where $\tilde{u}$ represents the periodic displacement field and $\tilde{\varepsilon }$ denotes the correlation between the microscopic ε and the PBCs.

**Figure 5:**
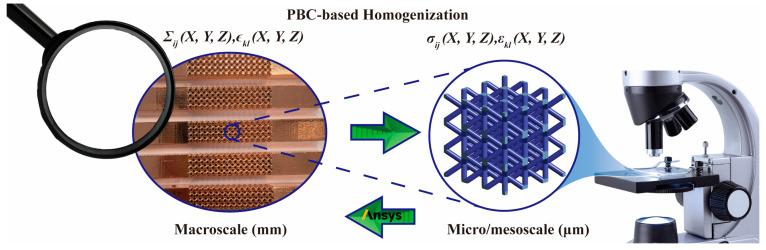
Multiscale analytic hierarchy in macroscale and micro/mesoscale domains.

Utilizing PBCs on the periodical surfaces of the copper-coated BCC lattice RVE in three orthogonal directions, $E_{lattice}$ is assessed with the applied u. Moreover, in the context of copper-coated lattice structures in this study, defining the RVE based on geometrical configuration is inappropriate due to internal voids inherent in lattice structures. Consequently, selecting an appropriate number of lattice cells for the RVE becomes essential to eliminate edge effects, a critical factor in determining structural mechanical properties at the micro/mesoscale [55,56,57]. To ascertain the minimum RVE unit size that exhibits size-independent equivalent mechanical properties, RVE models with six types of composed lattice unit sizes are considered, ranging from 1 × 1 × 1 to 6 × 6 × 6. Tetrahedral elements with quadratic interpolation mode (Solid187 element) are used to discretize the lattice unit base, chosen for their accuracy and adaptability to lattices with high geometrical complexity. Overall, as depicted in Figure 5, the multiscale analytical hierarchy of the coated lattices unveils the equivalent mapping from macroscale experiments to micro/mesoscopic RVE-based simulations. 

#### 2.2.2. FE Modeling of the Metal Coating and Lattice Bulk

The CAD models of the BCC lattice cells are established with a parameterized variable, d. The ratio η of struts accounting for the lattice cubic volume is determined by $\eta =V_{solid}/V_{cubic}$. For the numerical simulation of the metal coating, given that T is in the mesoscale and considerably smaller than d, its stiffness behavior is assumed to be membrane and bending [58]. Consequently, the coating film can be constructed with one-layer elements on top of the strut base, considering the stiffness behaviors of membrane and bending. The transverse shear stiffness $E_{trans}$ of the coating film is described as follows:

(3) $$E_{trans}=\left[\begin{array}{cc} kGT & 0\\ 0 & kGT \end{array}\right],$$

where k represents the shear-correction factor and *G* represents the shear modulus. Although the base-film contacting settings are neglected, the versatility of this coating modeling method in both linear and nonlinear elasticity implies its comprehensive use in diverse material models. Moreover, when discretizing the coating film based on solid elements, it is necessary to use tiny element sizes, which, in turn, would lead to a significant increase in element numbers. To strike a balance between computation efficiency and calculation precision, the one-layer element-based modeling method was chosen over others with advanced hybrid elements. 

## 3. Results and Discussion

### 3.1. Validation of the PBC-Based Multiscale Method and RVE Size Effect

**Figure 6:**
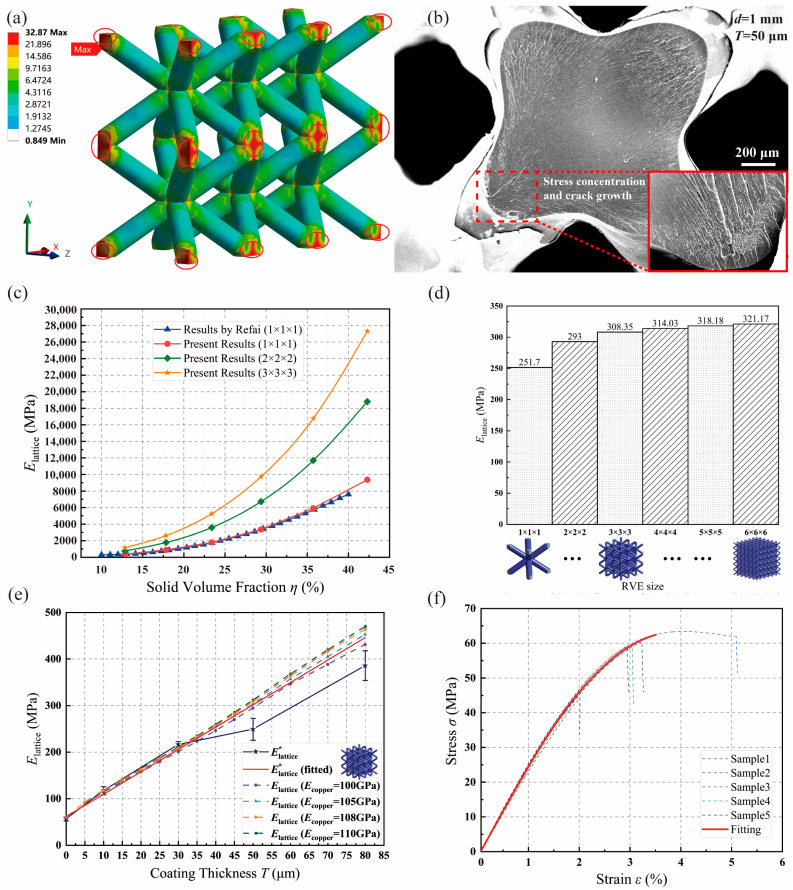
Mechanical characterization of $E_{lattice}$ using a PBC-based mesoscale explanation: (**a**) RVE-based lattice contour results of von Mises stress on a global scale; (**b**) SEM image of the cross-section scheme of the copper-coated lattice structure with d=1 mm andT=50 μm; (**c**) $E_{lattice}$ comparison based on RVE with ones obtained by Refai [50]; (**d**) impact of RVE size on $E_{lattice}$; (**e**) variations in $E_{lattice}$ and $E_{lattice}^{*}$ with T (d=0.6 mm); and (**f**) stress–strain curves of the resin matrix.

**Figure A1:**
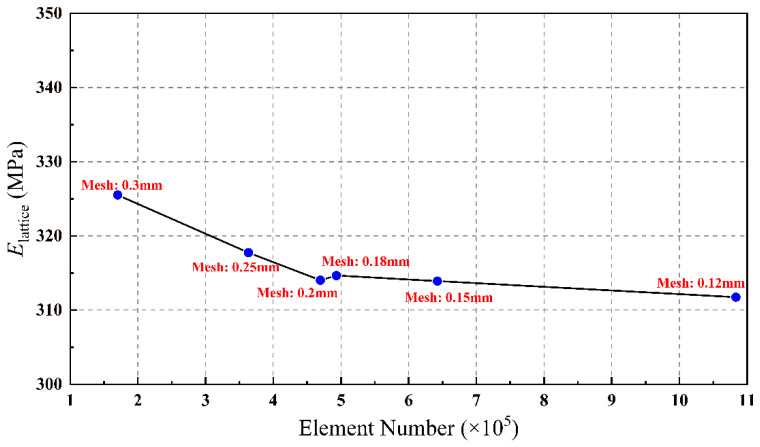
$E_{lattice}$ of the 4 × 4 × 4 copper-coated lattice RVE by different mesh sizes.

Taking into account the geometrical configuration of the lattice cell, the appropriate lattice cell number for an efficient and stable anticipation of the mechanical response is determined with the mesh convergence study depicted in Figure A1. Under the applied PBCs with a uniform u of 0.1 mm, the von Mises stress contour of the coated lattice structure with d=1 mm and T=50 μm is described in Figure 6a. The results showcase excellent compatibility between the RVE and the structural scale. Furthermore, a notable consistency is observed between the maximum stress distribution and the crack patterns revealed in the scanning electron microscope (SEM) graphs shown in Figure 6b. This validates the rationality of the multiscale method in assessing the lattice structure’s property. 

Moving on to the variation in $E_{lattice}$ with parameterized d, the results of the lattice unit model based on the proposed multiscale method are compared with the results of the 1 × 1 × 1 unit model by Refai [50]. Additionally, RVE models with the sizes of 2 × 2 × 2 and 3 × 3 × 3 are also evaluated. As illustrated in Figure 6c, a robust agreement is presented between the author’s results and the literature results when predicting the lattice’s effective structural property with the 1 × 1 × 1 unit model. This underscores the precision of homogenization and PBC-based multiscale analysis methods. However, the significant influence of RVE size on the structural evaluation is also revealed, indicating the inevitability of RVE size consideration for lattice structures. 

The significance of lattice cell numbers within a single RVE on $E_{lattice}$ is illustrated in detail using the coated lattice RVE. Figure 6d denotes a significant variation when the RVE dimension deviates from 1 × 1 × 1 to 3 × 3 × 3, while a relatively stable and converged trend is witnessed with the size of the RVE model exceeding 3 × 3 × 3. In other words, in contrast to the conventional RVE determination method used in composites, the RVE definition of metal-coated lattice structures should consider the size effect of the RVE. The existing mutual interference between coated lattice units cannot be neglected, and the $E_{lattice}$ assessed based on cells comprising multiple units is more stable than that based on a single unit. Considering the growth rate of element numbers with increased RVE unit dimension shown in Table 1, the suggested minimum unit size of the RVE model for metal-coated lattice structures is 3 × 3 × 3 for a more reasonable and efficient anticipation of equivalent mechanical properties based on homogenization theory. 

### 3.2. Property Determination of Resin Matrix and Copper Coating 

To mitigate the impact of copper film characterization on numerical analysis, we analyze the equivalent properties of the coated lattice structure using four correspondingly defined values of $E_{copper}$. As depicted in Figure 6e, the utilization of a 3 × 3 × 3 lattice RVE model with d=0.6 mm results in favorable result alignments in RVE-based multiscale evaluations, comparing well with the fitted global experiment results across all $E_{copper}$ values. Additionally, a quasi-linear relationship between T and $E_{lattice}$, as previously noted by Zheng et al. [21], is evident. With variations in T, changes in $E_{lattice}$ under diverse selections of $E_{copper}$, as well as the fitted $E_{lattice}^{*}$, are anticipated and detailed in detail in Table 2. This validates the effectiveness and accuracy of the proposed multiscale method for the mechanical characterization of the coated lattice. Furthermore, $E_{copper}$ is determined to be 105 GPa for subsequent analyses under the current electrodepositing technique, ensuring numerical precision.

Turning to the photopolymer matrix, as illustrated in Figure 6f, stress–strain curves for five dog-bone samples are obtained and further fitted to define the material properties of the polymer resin. Detailed information is presented in Table 3. Therefore, $E_{resin}$ of 2.65 GPa is utilized for subsequent numerical predictions. 

### 3.3. Characterization Interpretations of the Copper-Coated Lattice

#### 3.3.1. Elastic Tensile Stiffness

**Figure 7:**
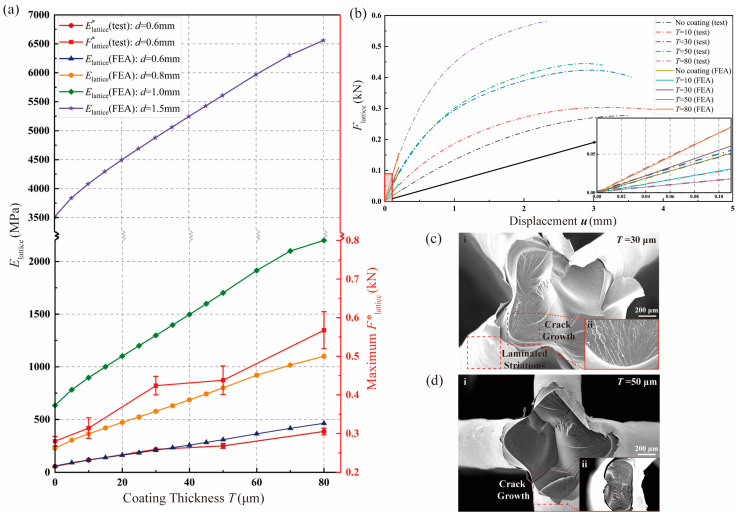
Elastic tensile stiffness characterizations of copper-coated lattice structures: (**a**) comparison between $E_{lattice}^{\;}$ and $E_{lattice}^{*}$ and factor analyses concerning the influences of T and d on $E_{lattice}^{\;}$ and maximum $F_{lattice}^{*}$; (**b**) comparison between $F_{lattice}^{\;}$ and $F_{lattice}^{*}$ and variations in ductility for coated lattice structures with different T values; (**c**) SEM image of coated lattice structures with T=30 μm under tensile loads including (i): an overview of the entire cross-section and (ii): a close-up view of crack positions; and (**d**) SEM graph of coated lattice structures with T=50 μm under tensile loads, including (i): an overview of the entire cross-section and (ii): a close-up view of crack positions.

Macroscopic evaluations of structural characteristics are carried out, focusing on the impact of T on both the maximum $F_{lattice}^{*\;}$ and $E_{lattice}^{*}$, with d set at 0.6 mm. As illustrated in Figure 7a, the loading capacity represented by $F_{lattice}^{*\;}$ demonstrates an increasing trend with higher T, signifying an improvement in structural strength. Regarding structural stiffness, $E_{lattice}^{*}$ also exhibits a noteworthy enhancement with the rise in T. The greater material density of the copper coating film, compared with the resin matrix, effectively boosts structural stiffness. Additionally, there is a strong agreement between the 3 × 3 × 3 lattice RVE-based $E_{lattice}$ and the macroscale structure-based $E_{lattice}^{*}$, validating the efficacy and accuracy of the proposed multiscale method. Building on this foundation, variations in $E_{lattice}$ concerning the design variables of d and T are further explored at the RVE level in Figure 7a. $E_{lattice}$ demonstrates a substantial improvement with increased T under each condition of d. Furthermore, it is rational to observe that the higher η resulting from the larger d contributes to the superior performance of the metal-coated lattice structure. Importantly, the increasing rate of $E_{lattice}$ over T is positively correlated to d, indicating that the enhancement in $E_{lattice}$ is more efficient with higher η under the same extent of T advancement. This holds significance for future designs of metal-coated lattices with high elastic tensile stiffness.

**Figure A2:**
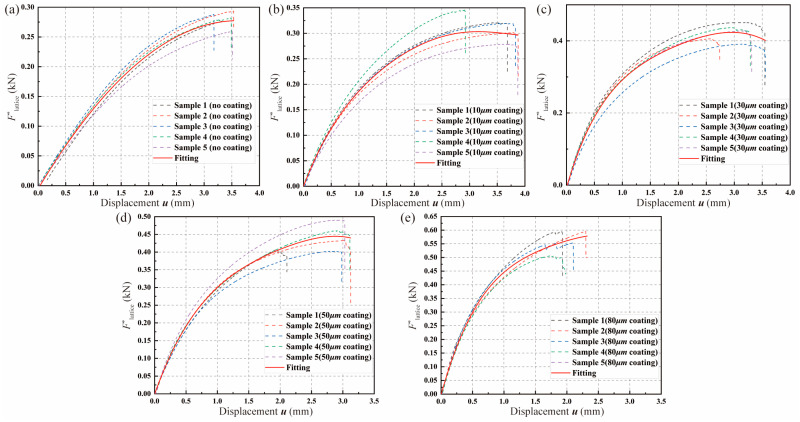
The influence of T on structural strength and ductility by experimental force–displacement results with (**a**) pure resin lattice matrix, (**b**) 10 μmcoated lattice, (**c**) 30 μmcoated lattice, (**d**) 50 μmcoated lattice, and (**e**) 80 μmcoated lattice.

Concerning structural ductility, force–displacement relationships of the coated lattice structures with varying values of T are examined, with d defined as 0.6 mm. Utilizing test results from the lattice samples depicted in Figure A2, force–displacement dot-dash lines are fitted from samples with five T values, as illustrated in Figure 7b. This analysis unveils an overall enhancement in the ductility of the coated lattice structure compared with the resin lattice matrix. Optimal ductility performance is observed at T=10 μm, with a declining trend for larger T. While copper generally exhibits superior ductile performance compared with resin, increased T does not consistently enhance structural ductility. Surprisingly, the relatively thick coating, compared with the curve result of the pure resin lattice matrix, negatively impacts the overall structure’s ductility. For structural optimization, considering a balanced improvement in structural strength and ductility, 30 μm coated lattice structures achieve optimal performance. Moreover, within the elastic deformation range (u≤0.1 mm) shown in the detailed view in Figure 7b, the RVE-based force–displacement anticipation lines of coated BCC lattices align closely with fitted test result lines under various T conditions. This alignment highlights the precision and applicability of the proposed multiscale method in evaluating applied force during elastic deformation.

For a more detailed explanation of the optimal balanced performance observed in the 30 μm coated lattice structure, the fracture surfaces of the 30 μm and 50 μm coated lattice structures are analyzed with SEM images. As shown in Figure 7c(ii),d(ii), striated fracture surfaces are exhibited on the resin matrix of both coated lattice structures with diverse T, and cracks emerge from the interface of adjacent layers, which is reasonable, owing to the layered print mechanism of 3D printing [26]. Specifically, the laminated striations on the coating film of the 30 μm coated lattice structure in Figure 7c(i) reveal the torsional effect that occurs during tension. However, for the coated lattices with T=50 μm in Figure 7d(i), there is no distinct torsional trace on the coating surface. Therefore, the untraditional torsion effect generated during the tension test of the 30 μmcoated lattice structure illustrates its impressive performances in both strength and ductility. 

#### 3.3.2. Elastic Bending Stiffness

**Figure 8:**
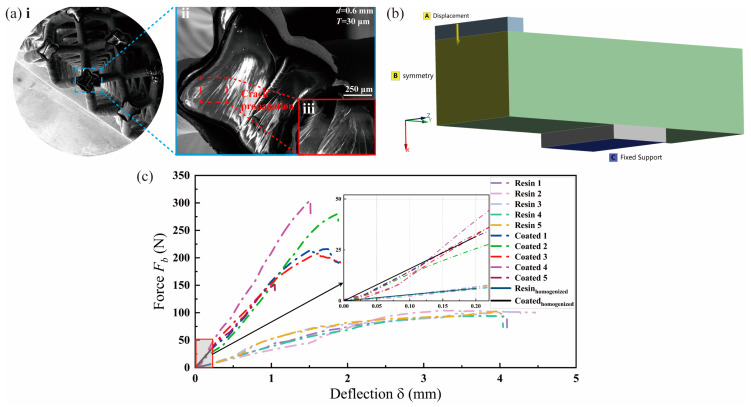
Elastic bending stiffness characterizations of copper-coated lattice structures: (**a**) SEM images displaying fracture cross-sections of the coated lattice structures under bending loads, with (i): an overview of the entire cross-section, (ii): a close-up view revealing crack patterns, and (iii): river-like fracture crack patterns; (**b**) numerical assessment of bending stiffness utilizing homogenized material properties; and (**c**) evaluations of $k_{b}$ for the resin and copper-coated lattice structures, comparing experimental and numerical methods.

Regarding $k_{b}$ of the coated lattice structure, the numerical evaluations are conducted for both resin and copper-coated lattice structures, and the results are compared with the tests. Initial mechanical interpretations of the coated lattices’ bending properties are performed using SEM images of the fracture cross-sections of the coated lattice structures. As described in Figure 8a(i), in terms of coating shedding, the copper coating electroplating technique is shown to be effective and can withstand multi-load conditions of both bending and stretching. In the enlarged view of the fracture surface in Figure 8a(ii), fracture cracks exhibit river-like patterns, and their depths are much deeper than those deriving from the tensile test in Figure 7c, revealing the better plasticity performance and potential of the copper-coated lattice structure under bending loads than under tensile loads. Moreover, coated lattice structures present edge-to-edge crack initiation and propagation characteristics under bending, while point-to-center ones are observed under stretching. This reasonably reflects the stress state differences between the two typical loading conditions. In other words, the difference in the fracture failure pattern in the coated lattice structure under stretching and bending is attributed to the stress diversity induced by external loads instead of other factors, such as printing directions. 

Following this, the $k_{b}$ of both the resin and copper-coated lattice structures is numerically evaluated using solid FE models with homogenized material properties. Figure 8b outlines the established model and boundary conditions, considering the geometrical and loading symmetry of the lattice structures’ bending experiments, as shown in Figure 3d. In this case, d and T are defined as 0.6 mm and 30 μm, respectively. Thus, the homogenized values of $E_{lattice}$ for the resin and copper-coated lattice structures are correspondingly determined as 60 MPa and 205 MPa, respectively, as detailed in Table 2. 

Figure 8c visually presents the impact of the copper coating, showcasing a significant 5-fold improvement in $k_{b}$ compared with the resin lattice matrix. The detailed view of the elastic displacement region (δ ≤ 0.2 μm) not only validates the rationale and precision of the homogenized material-based numerical method based on $k_{b}$ comparisons for both conditions of the lattice structures—with or without copper film—but also underscores the efficiency and precision of the multiscale method-based evaluations of $E_{lattice}$. This approach offers a streamlined and resource-efficient means of defining the mechanical properties of metal-coated lattices, eliminating the need for time-consuming and resource-intensive experimental setups. 

## 4. Conclusions

This study introduces a lattice PBC-based multiscale evaluation method to efficiently predict the mechanical properties of copper-coated lattice structures at the RVE scale. The key conclusions derived from this research are as follows:

1. Feasibility Validation: The proposed method’s feasibility is substantiated by comparing numerical and experimental results for both elastic tensile stiffness $E_{lattice}$ and bending stiffness $k_{b}$. The accuracy in assessing these properties demonstrates the efficacy of the lattice PBC-based multiscale approach.
2. Homogenized Model for Numerical Analysis: The utilization of a solid homogenized model in numerical analysis, where $E_{lattice}$ is defined through the proposed multiscale method, proves effective in accurately evaluating $k_{b}$. This streamlined approach offers a precise means to comprehend the elastic behavior of coated lattice structures without the need for intricate lattice cell discretization.
3. Impact of Design Parameters: The influence of design parameters on the mechanical characteristics of copper-coated lattice structures is apparent. Specifically, $E_{lattice}$ for coated lattices increases with greater coating thickness and strut diameter. The presence of a metal coating significantly enhances the structural $k_{b}$. Bending loads exploit the structural plasticity potential of coated lattices more thoroughly compared with pure resin lattice matrices. The diversity in structural failure patterns is primarily attributed to loading differences. These findings provide valuable insights for future coated lattice design and optimization efforts.

## Figures and Tables

**Table 1:** Discretized element numbers of RVEs containing diverse cell numbers.

RVE Size	1 × 1 × 1	2 × 2 × 2	3 × 3 × 3	4 × 4 × 4	5 × 5 × 5	6 × 6 × 6
Element numbers	7101	54,258	187,971	469,571	942,533	1,632,026

**Table 2:** Comparisons between $E_{lattice}$ and $E_{lattice}^{*}$.

***T*** (μm)	$E_{lattice}$ (MPa)				***T*** (μm)	$E_{lattice}^{*}$ (MPa)	***Error*** (MPa)	***T*** (μm)	Fitted $E_{lattice}^{*}$ (MPa)
	***E**_**copper**_* = 100 GPa	$E_{copper}$ = 105 GPa	$E_{copper}$ = 108 GPa	$E_{copper}$ = 110 GPa					
0	60.074	60.074	60.074	60.074	0	56.61	4.44	0	61.251
5	90.048	91.262	91.983	92.459	10	117.25	8.52	5.04	85.488
10	113.99	116.01	117.2	117.99	30	216.36	6.26	10.01	109.340
15	135.98	138.71	140.33	141.4	50	249.20	23.47	15.05	133.576
20	157.38	160.83	162.88	164.23	80	385.98	31.64	20.02	157.429
25	178.82	183.03	185.52	187.16				25.06	181.665
30	200.64	205.63	208.6	210.56				30.03	205.517
35	223.02	228.85	232.32	234.61				35.07	229.754
40	246.08	252.8	256.8	259.44				40.04	253.606
45	269.9	277.56	282.12	285.15				45.00	277.459
50	294.54	303.2	308.35	311.77				50.05	301.695
60	346.45	357.25	363.68	367.96				55.01	325.547
70	388.49	404.78	416.24	419.56				70.07	397.873
80	430.89	452.64	463.75	469.75				80.00	445.577

**Table 3:** Material properties of the resin and copper film.

Resin Matrix			Copper Film
$E_{resin}$ of samples (GPa)	Mean of $E_{resin}$ (GPa)	Fitting of $E_{resin}$ (GPa)	$E_{copper}$ (GPa)
2.52, 2.82, 2.73, 2.46, 2.59	2.62	2.65	105

## Data Availability

The datasets generated during and analyzed during the current study are available from the corresponding author upon reasonable request.

## Appendix A. Mesh Convergence Analysis

To mitigate the size effect of meshed elements, a mesh convergence analysis is performed using a 4 × 4 × 4 copper-coated lattice RVE. As shown in Figure A1, $E_{lattice}$ exhibits fluctuations within 0.72% when the mesh size surpasses 0.2 mm. The element numbers corresponding to mesh sizes of 0.18 mm, 0.15 mm, and 0.12 mm are more than 1.05, 1.37, and 2.31 times that of the 0.2 mm size, respectively. Therefore, considering the balance between accuracy and computational cost, a mesh size of 0.2 mm is deemed suitable for the factor analysis of various coated lattice RVEs.

## Appendix B. Strength and Ductility Analysis of Diverse-Coated Lattices

See Figure A2.

## References

[1] Zhang L., Song B., Fu J.J., Wei S.S., Yang L., Yan C.Z., Li H., Gao L., Shi Y.S. (2020). Topology-optimized lattice structures with simultaneously high stiffness and light weight fabricated by selective laser melting: Design, manufacturing and characterization. J. Manuf. Process..

[2] Hamzehei R., Rezaei S., Kadkhodapour J., Anaraki A.P., Mahmoudi A. (2020). 2D triangular anti-trichiral structures and auxetic stents with symmetric shrinkage behavior and high energy absorption. Mech. Mater..

[3] Sorrentino A., Castagnetti D. (2022). Negative Poisson’s ratio lattice for designing vertebral biomaterials. Mech. Adv. Mater. Struct..

[4] Baertsch F., Ameli A., Mayer T. (2021). Finite-Element Modeling and Optimization of 3D-Printed Auxetic Reentrant Structures with Stiffness Gradient under Low-Velocity Impact. J. Eng. Mech..

[5] Luo C., Han C.Z., Zhang X.Y., Zhang X.G., Ren X., Xie Y.M. (2021). Design, manufacturing and applications of auxetic tubular structures: A review. Thin-Walled Struct..

[6] Wu W., Song X., Liang J., Xia R., Qian G., Fang D. (2018). Mechanical properties of anti-tetrachiral auxetic stents. Compos. Struct..

[7] Zhao Z., Li J., Wei Y., Yu T. (2022). Design and properties of graded polyamide12/hydroxyapatite scaffolds based on primitive lattices using selective laser sintering. J. Mech. Behav. Biomed. Mater..

[8] Veloso F., Miranda D., Morais P., Torres H.R., Oliveira B., Correia-Pinto J., Pinho A.C., Vilaça J.L. (2022). Study of the compression behavior of functionally graded lattice for customized cranial remodeling orthosis. J. Mech. Behav. Biomed. Mater..

[9] Ngo T.D., Kashani A., Imbalzano G., Nguyen K.T.Q., Hui D. (2018). Additive manufacturing (3D printing): A review of materials, methods, applications and challenges. Compos. B Eng..

[10] McMillan M., Jurg M., Leary M., Brandt M. (2015). Programmatic lattice generation for additive manufacture. Procedia Technol..

[11] Tamburrino F., Graziosi S., Bordegoni M. (2018). The design process of additively manufactured mesoscale lattice structures: A review. J. Comput. Inf. Sci. Eng..

[12] Ali M.N., Rehman I.U. (2011). An Auxetic structure configured as oesophageal stent with potential to be used for palliative treatment of oesophageal cancer; development and in vitro mechanical analysis. J. Mater. Sci. Mater. Med..

[13] Yang L., Harrysson O., West H., Cormier D. (2015). Mechanical properties of 3D re-entrant honeycomb auxetic structures realized via additive manufacturing. Int. J. Solids Struct..

[14] Vilardell A.M., Takezawa A., Du Plessis A., Takata N., Krakhmalev P., Kobashi M., Albu M., Kothleitner G., Yadroitsava I., Yadroitsev I. (2021). Mechanical behavior of in-situ alloyed Ti6Al4V (ELI)-3 at.% Cu lattice structures manufactured by laser powder bed fusion and designed for implant applications. J. Mech. Behav. Biomed. Mater..

[15] Kang K.-J. (2015). Wire-woven cellular metals: The present and future. Prog. Mater. Sci..

[16] Plocher J., Mencattelli L., Narducci F., Pinho S. (2021). Learning from nature: Bio-inspiration for damage-tolerant high-performance fibre-reinforced composites. Compos. Sci. Technol..

[17] Aghajani S., Wu C., Li Q., Fang J. (2023). Additively manufactured composite lattices: A state-of-the-art review on fabrications, architectures, constituent materials, mechanical properties, and future directions. Thin-Walled Struct..

[18] Hunt C.J., Morabito F., Grace C., Zhao Y., Woods B.K.S. (2022). A review of composite lattice structures. Compos. Struct..

[19] Nazir A., Gokcekaya O., Billah K.M.M., Ertugrul O., Jiang J., Sun J., Hussain S. (2023). Multi-material additive manufacturing: A systematic review of design, properties, applications, challenges, and 3D printing of materials and cellular metamaterials. Mater. Des..

[20] Garcia-Taormina A.R., Alwen A., Schwaiger R., Hodge A.M. (2021). A review of coated nano- and micro-lattice materials. J. Mater. Res..

[21] Yuan H., Zhang W. A Novel Hedgehog-Inspired Pin-Array Robot Hand with Multiple Magnetic Pins for Adaptive Grasping. Proceedings of the Intelligent Robotics and Applications: 12th International Conference (ICIRA 2019).

[22] Barri K., Jiao P., Zhang Q., Chen J., Wang Z.L., Alavi A.H. (2021). Multifunctional meta-tribomaterial nanogenerators for energy harvesting and active sensing. Nano Energy.

[23] He L., Wang P., Yang J., Fan K., Zhang H., Zhang L., Jiang M., Chen X., Chen Z., Chen M. (2023). Smart Lattice Structures with Self-Sensing Functionalities via Hybrid Additive Manufacturing Technology. Micromachines.

[24] Schroer A., Wheeler J.M., Schwaiger R. (2018). Deformation behavior and energy absorption capability of polymer and ceramic-polymer composite microlattices under cyclic loading. J. Mater. Res..

[25] Diamantopoulou M., Roth C.C., Tancogne-Dejean T., Lauener C.M., Mohr D. (2022). Ceramic/polymer microlattices: Increasing specific energy absorption through sandwich construction. Extrem. Mech. Lett..

[26] Mieszala M., Hasegawa M., Guillonneau G., Bauer J., Raghavan R., Frantz C., Kraft O., Mischler S., Michler J., Philippe L. (2017). Micromechanics of Amorphous Metal/Polymer Hybrid Structures with 3D Cellular Architectures: Size Effects, Buckling Behavior, and Energy Absorption Capability. Small.

[27] Kao C.-T., Lin C.-C., Chen Y.-W., Yeh C.-H., Fang H.-Y., Shie M.-Y. (2015). Poly (dopamine) coating of 3D printed poly (lactic acid) scaffolds for bone tissue engineering. Mater. Sci. Eng. C.

[28] Xiao R., Feng X., Fan R., Chen S., Song J., Gao L., Lu Y. (2020). 3D printing of titanium-coated gradient composite lattices for lightweight mandibular prosthesis. Compos. B Eng..

[29] Ng H.W., Gan Z. (2005). A finite element analysis technique for predicting as-sprayed residual stresses generated by the plasma spray coating process. Finite Elem. Anal. Des..

[30] Bencheikh I., Bilteryst F., Nouari M. (2017). Modelling of the thermomechanical behaviour of coated structures using single and multi-level-set techniques coupled with the eXtended Finite Element Method. Finite Elem. Anal. Des..

[31] Yilmaz K.B., Çömez I., Güler M.A., Yildirim B. (2019). Analytical and finite element solution of the sliding frictional contact problem for a homogeneous orthotropic coating-isotropic substrate system. ZAMM-J. Appl. Math. Mech./Z. Angew. Math. Mech..

[32] Jahedi R., Adibnazari S., Farrahi G.H. (2015). Performance analysis of functionally graded coatings in contact with cylindrical rollers. Adv. Mech. Eng..

[33] Sobol B., Soloviev A., Krasnoschekov A. (2015). The transverse crack problem for elastic bodies stiffened by thin elastic coating. ZAMM-J. Appl. Math. Mech./Z. Angew. Math. Mech..

[34] Salari-Sharif L., Schaedler T.A., Valdevit L. (2018). Hybrid Hollow Microlattices with Unique Combination of Stiffness and Damping. J. Eng. Mater. Technol. Trans. ASME.

[35] Song J., Gao L., Cao K., Zhang H., Xu S., Jiang C., Surjadi J.U., Xu Y., Lu Y. (2018). Metal-coated hybrid meso-lattice composites and their mechanical characterizations. Compos. Struct..

[36] Greco F., Leonetti L., Pranno A., Rudykh S. (2020). Mechanical behavior of bio-inspired nacre-like composites: A hybrid multiscale modeling approach. Compos. Struct..

[37] Christoff B.G., Almeida J.H.S., Cardoso E.L., Tita V. (2023). A multiscale topology optimisation framework for hollow spheres as cellular materials. Eng. Struct..

[38] Jimenez Abarca M., Darabi R., de Sa J.C., Parente M., Reis A. (2023). Multi-scale modelling modeling for prediction of residual stress and distortion in Ti-6Al-4V Ti–6Al–4V semi-circular thin-walled parts additively manufactured by laser powder bed fusion (LPBF). Thin-Walled Struct..

[39] Kurukuri S., Eckardt S. (2004). A Review of Homogenization Techniques for Heterogeneous Materials.

[40] Ostoja-Starzewski M. (2006). Material spatial randomness: From statistical to representative volume element. Probabilistic Eng. Mech..

[41] Ptochos E., Labeas G. (2012). Elastic modulus and Poisson’s ratio determination of micro-lattice cellular structures by analytical, numerical and homogenisation methods. J. Sandw. Struct. Mater..

[42] He L., Wang P., Wang L., Chen M., Liu H., Li J. (2023). Multifunctional Polymer-Metal Lattice Composites via Hybrid Additive Manufacturing Technology. Micromachines.

[43] Shacham-Diamand Y., Osaka T., Okinaka Y., Sugiyama A., Dubin V. (2015). 30 Years of electroless plating for semiconductor and polymer micro-systems. Microelectron. Eng..

[44] Li J., Wang Y., Xiang G., Liu H., He J. (2019). Hybrid Additive Manufacturing Method for Selective Plating of Freeform Circuitry on 3D Printed Plastic Structure. Adv. Mater. Technol..

[45] (2022). Standard Test Method for Tensile Properties of Plastics.

[46] Zhou Y., Yang C.S., Chen J.A., Ding G.F., Ding W., Wang L., Wang M.J., Zhang Y.M., Zhang T.H. (2004). Measurement of Young’s modulus and residual stress of copper film electroplated on silicon wafer. Thin Solid Films.

[47] Hong S.H., Kim K.S., Kim Y.M., Hahn J.H., Lee C.S., Park J.H. (2005). Characterization of elastic moduli of Cu thin films using nanoindentation technique. Compos. Sci. Technol..

[48] Zak S., Trost C.O.W., Kreiml P., Cordill M.J. (2022). Accurate measurement of thin film mechanical properties using nanoindentation. J. Mater. Res..

[49] Dixit P., Xu L., Miao J., Pang J.H.L., Preisser R. (2007). Mechanical and microstructural characterization of high aspect ratio through-wafer electroplated copper interconnects. J. Micromech. Microeng..

[50] Refai K., Montemurro M., Brugger C., Saintier N. (2020). Determination of the effective elastic properties of titanium lattice structures. Mech. Adv. Mater. Struct..

[51] Mao H., Rumpler R., Göransson P. (2020). An inverse method for characterisation of the static elastic Hooke’s tensors of solid frame of anisotropic open-cell materials. Int. J. Eng. Sci..

[52] Belardi V.G., Trupiano S., Fanelli P., Vivio F. (2024). Overall elastic characterization of equivalent FE models for aluminum foams through computational homogenization approach and genetic algorithm optimization. Eur. J. Mech. A/Solids.

[53] Li C., Chen L., Qiao L. (2012). RVE Based Numerical Evaluation on Effective Mechanical Properties of Composite with Randomly Distributed Multi-Phase Inclusions. Adv. Mat. Res..

[54] Wang L., Chen M., Chen G., Luo T., Liu F. (2023). Loading capacity prediction of the auxetic tubular lattice structures by multiscale shakedown analysis. Compos. Struct..

[55] Herrnböck L., Steinmann P. (2022). Homogenization of fully nonlinear rod lattice structures: On the size of the RVE and micro structural instabilities. Comput. Mech..

[56] Moeini M., Begon M., Lévesque M. (2022). Numerical homogenization of a linearly elastic honeycomb lattice structure and comparison with analytical and experimental results. Mech. Mater..

[57] Wang X., Zhu L., Sun L., Li N. (2021). Optimization of graded filleted lattice structures subject to yield and buckling constraints. Mater. Des..

[58] Wang X., Yuan F., Chen M., He J., Wang P., Yu Y., Li J. (2019). Investigation on mechanical characterizations of metal-coated lattice structure. Sustainable Buildings and Structures: Building a Sustainable Tomorrow.

